# Piroxicam and intracavitary platinum-based chemotherapy for the treatment of advanced mesothelioma in pets: preliminary observations

**DOI:** 10.1186/1756-9966-27-6

**Published:** 2008-05-19

**Authors:** Enrico P Spugnini, Stefania Crispi, Alessandra Scarabello, Giovanni Caruso, Gennaro Citro, Alfonso Baldi

**Affiliations:** 1 S.A.F.U. Department, Regina Elena Cancer Institute –, Rome –, Italy; 2 Gene Expression Core, – Human Molecular Genetics Laboratory, Institute of Genetics and Biophisycs, CNR, Naples, Italy; 3 Istituto Dermatologico San Gallicano, Rome, Italy; 4 Department of Biochemistry, section of Pathology, Second University of Naples, Italy

## Abstract

Malignant Mesothelioma is an uncommon and very aggressive tumor that accounts for 1% of all the deaths secondary to malignancy in humans. Interestingly, this neoplasm has been occasionally described in companion animals as well. Aim of this study was the preclinical evaluation of the combination of piroxicam with platinum-based intracavitary chemotherapy in pets. Three companion animals have been treated in a three years period with this combination. Diagnosis was obtained by ultrasonographic exam of the body cavities that evidenced thickening of the mesothelium. A surgical biopsy further substantiated the diagnosis. After drainage of the malignant effusion from the affected cavity, the patients received four cycles of intracavitary CDDP at the dose of 50 mg/m^2 ^every three weeks if dogs or four cycles of intracavitary carboplatin at the dose of 180 mg/m^2 ^(every 3 weeks) if cats, coupled with daily administration of piroxicam at the dose of 0.3 mg/kg. The therapy was able to arrest the effusion in all patients for variable remission times: one dog is still in remission after 3 years, one dog died of progressive disease after 8 months and one cat died due to progressive neoplastic growth after six months, when the patient developed a mesothelial cuirass. The combination showed remarkable efficacy at controlling the malignant effusion secondary to MM in our patients and warrants further investigations.

## Introduction

Malignant mesothelioma (MM) is a rare, highly aggressive tumor, accounting for less than 1% of all cancer deaths in the world [1]. This neoplasm arises from the surface serosal cells of the pleural (> 90% of cases), peritoneal, and pericardial cavities and from the tunica vaginalis of the testis [2]. Several epidemiological and clinicopathological studies have shown a correlation between exposure to asbestos and development of pleural mesothelioma [3]; nevertheless, the exact mechanism whereby asbestos induces the mesothelioma is still unknown [4,5]. Interestingly, recent studies have proposed a role for Simian Virus 40 large T antigen (SV40-Tag) in the pathogenesis of human mesothelioma, but there are discordant opinions in the scientific literature [6-8] Due to the low incidence of the disease, only few randomized studies have been performed [reviewed in [9]]. The prognosis is generally poor with a reported median survival of 4 to 12 months in either untreated or treated (surgery, radiotherapy, or chemotherapy) patients [10]. Moreover, mesothelioma has proven resistant to classical chemotherapeutic and radiation regimes and the natural history has not been influenced by standard therapy [11]. Various drugs have been tested in different combinations so far; among the most commonly employed are doxorubicin, cyclophosphamide, cisplatin (CDDP), carboplatin, gemcitabine, and pemetrexed [11-15]. Of interest, the combination of these drugs, with the exception of pemetrexed, does not appear to provide any clear advantage over mono-therapy [9]. Therefore, there is need of more effective therapies, possibly based on the most recent discoveries on the molecular events involved in mesothelioma pathogenesis and progression. Our research group has recently demonstrated that piroxicam, a commonly used NSAID and COX-inhibitor, has the capability to potentiate the chemotherapic effects of cisplatin on mesothelioma cells, both *in vitro *and *in vivo *[16]. This tumor has been infrequently reported in dogs [17] and even more rarely described in cats [18-24]. Moreover, treatment has been limited to palliation with corticosteroids or repeated drainage of malignant effusion, or intracavitary platinum coumpounds [24,25].

A recent study in a mouse model has suggested a potential synergic action between piroxicam and platinum compounds for the treatment of orthotopic mesothelioma xenografts [16].

## Materials and methods

Privately owned companion animals (two dogs and one cat) with MM were enrolled to receive the combination of platinum compounds and piroxicam.

MM was diagnosed by means of ultrasonographic examination confirmed by histopathologically examination of biopsy specimens.

### Treatment protocol

After tumor biopsy or debulking, dogs received daily piroxicam at the dose of 0.3 mg/kg PO for 4 months and CDDP at the dose of 50 mg/m^2 ^every three weeks intraperitoneally, for a total of four doses. The patients had a complete blood cell count, biochemical profile and urinalysis performed 7 days after each chemotherapy. A ultrasonographic exam was performed on a monthly basis to check for effusion secondary to malignancy.

The cat was treated with injectable piroxicam, (in order to better dose the drug), given at the dose of 0.3 mg/kg SC every other day [26] for 4 months and carboplatin at the dose of 180 mg/m^2 ^(every 3 weeks) for a total of four doses. Carboplatin was chosen since systemic administration of CDDP causes fatal pulmonary edema in cats [26]. The patient had a complete blood cell count, biochemical profile and urinalysis performed 7 days after each chemotherapy. A ultrasonographic exam was performed on a monthly basis to check for effusion secondary to malignancy.

## Results

**Figure 1:**
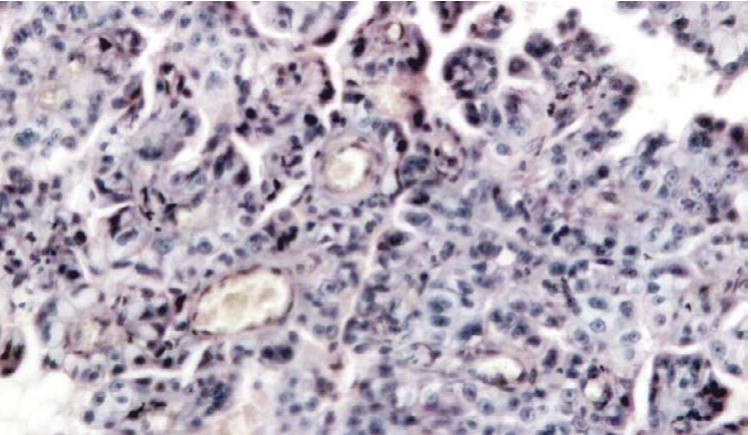
Malignant diffuse mesothelioma of epithelial type, forming dense sheets of tumor cells (Hematoxylin/Eosin, Original magnification 10 × 40).

All the enrolled patients well tolerated the treatment protocol without significant side effects that were limited to 1 episode of hematochezia in a dog and in the cat, requiring one week discontinuation of the piroxicam therapy. All the patients had cavitary effusion at the time of presentation: abdominal centesis lead to the removal of 180 ml of fluid as an average. Grossly, the fluid had a yellowish appearance with pink streaks and with occasional filaments of fibrin, microscopically the sample resulted highly cellular showed mesothelial cells isolated or gathered to form clusters, with coarse cromatin, presence of binucleate cells and nuclei of variable size with prominent and large nucleoli (not shown). Abdominal ultrasonography showed thickening of the abdominal serosal surfaces (not shown). Explorative surgery was performed to perform a tumor debulking in a dog and to collect biopsies in the other patients. Histological examination revealed for the two dogs a mixed mesothelioma while the cat had an epithelioid mesothelioma. In figure 1 the histoligical aspect of the epitheliod mesothelioma of the cat is depicted. One of the two dogs is still disease free after debulking and systemic therapy at 2 years, the other dog and the cat had a reduction of the fluid production by 90% as confirmed by sequential centesis, but developed progressive disease that let to organ strangulation after 8 and 6 months, respectively. Table 1 summarizes the patients' characteristics and follow-up.

**Table 1:** Individual data and response to therapy in 3 pets with Malignant Mesothelioma

**Species**	**Age**	**Location**	**Response**	**Outcome**
Syberian husky	9	peritoneum	24 months+	NED
Mixed Breed	11	peritoneum	8 months	Dead, PD
DSH	13	peritoneum	6 months	Dead, PD

DSH = domestic short hair, NED = no evidence of disease, PD = progressive disease

## Discussion

As with humans, pets cannot be cured of MM [17]. As with humans with down-staged MM at the time of diagnosis, surgical excision can only be rarely performed in pets [17]. The only exception to this rule may be pericardial MM, where successful palliation can sometimes be achieved by total or partial pericardiectomy, relieving the hemodynamic problems caused by tamponade [27,28]. Systemic chemotherapy using mitoxantrone or doxorubicin has been limited to case reports or to small series. Its effectiveness has been limited when used as single modality, while its use as an adjunct to cytoreduction (especially pericardiectomy) has led to reports of prolonged tumour control [29-31]. Probably the best use of chemotherapy for canine mesothelioma is still confined to palliation for advanced disease by means of intracavitary instillation. This approach is well tolerated and is capable of significantly decrease the amount of fluid released by the mesothelioma, furthermore it seems to induce tumor shrinking for a limited amount of time. A review of the literature showed that, in a small cohort of 6 dogs, treatment with intracavitary cisplatin resulted in survival times approaching one year in 5 patients [25]. Unfortunately the low penetration of the drug within the mesothelial layers (2–3 mm in depth) limits its usefulness in case of bulky disease. The use of other drugs such as tetracycline, talk, or bleomycin (Spugnini EP, personal observation) to induce pleurodesis has been unrewarding [32,33]. Our protocol showed to be highly tolerable by our patients and proved to successfully palliate the malignant effusion secondary to MM in two pets and was capable to induce a long term control in the third. Interestingly, none of the dogs had haematological or gastrointestinal toxicities, in particular vomiting that is one of the limiting factors to the use of cisplatin in dogs. Also the cat tolerated the therapy without side effects.

In conclusion, considering that there are not established chemotherapy protocols for pets with MM, the combination of intracavitary platinum compounds coupled with piroxicam should be further evaluated, also in view of the many similarities shared by canine and feline MM with that of humans. In fact, it has been shown in several research articles that veterinary cancer patients should be considered valuable models for the study of novel approaches such as chemosensitizers, immune modulators, imaging studies and new delivery systems for loco-regional therapy [34]. However, results should be evaluated with caution since it's likely that MM has a more benign biological course in dogs than it does in people and this has to be considered in evaluating uncontrolled case reports of 1 year survival in the dog.

## Authors' contributions

EPS performed the experiments and wrote the paper together with AB. SC, AS and GC participated in the design of the study and performed the histological staining. GC conceived of the study, and participated in its design and coordination. All authors read and approved the final manuscript.
